# A Quartz Crystal Microbalance, Which Tracks Four Overtones in Parallel with a Time Resolution of 10 Milliseconds: Application to Inkjet Printing

**DOI:** 10.3390/s20205915

**Published:** 2020-10-20

**Authors:** Christian Leppin, Sven Hampel, Frederick Sebastian Meyer, Arne Langhoff, Ursula Elisabeth Adriane Fittschen, Diethelm Johannsmann

**Affiliations:** 1 Institute of Physical Chemistry, Clausthal University of Technology, Arnold-Sommerfeld-Str. 4, D-38678 Clausthal-Zellerfeld, Germany; christian.leppin@tu-clausthal.de (C.L.); frederick.sebastian.meyer@tu-clausthal.de (F.S.M.); arne.langhoff@tu-clausthal.de (A.L.); 2 Institute of Inorganic and Analytical Chemistry, Clausthal University of Technology, Arnold-Sommerfeld-Str. 4, D-38678 Clausthal-Zellerfeld, Germany; sven.hampel@tu-clausthal.de (S.H.); ursula.fittschen@tu-clausthal.de (U.E.A.F.)

**Keywords:** inkjet printing, quartz crystal microbalance, QCM, fast QCM, picoliter-dosing, microfluidics, droplet-based microfluidics

## Abstract

A quartz crystal microbalance (QCM) is described, which simultaneously determines resonance frequency and bandwidth on four different overtones. The time resolution is 10 milliseconds. This fast, multi-overtone QCM is based on multi-frequency lockin amplification. Synchronous interrogation of overtones is needed, when the sample changes quickly and when information on the sample is to be extracted from the comparison between overtones. The application example is thermal inkjet-printing. At impact, the resonance frequencies change over a time shorter than 10 milliseconds. There is a further increase in the contact area, evidenced by an increasing common prefactor to the shifts in frequency, Δ*f*, and half-bandwidth, ΔΓ. The ratio ΔΓ/(−Δ*f*), which quantifies the energy dissipated per time and unit area, decreases with time. Often, there is a fast initial decrease, lasting for about 100 milliseconds, followed by a slower decrease, persisting over the entire drying time (a few seconds). Fitting the overtone dependence of Δ*f*(*n*) and ΔΓ(*n*) with power laws, one finds power-law exponents of about 1/2, characteristic of semi-infinite Newtonian liquids. The power-law exponents corresponding to Δ*f*(*n*) slightly increase with time. The decrease of ΔΓ/(−Δ*f*) and the increase of the exponents are explained by evaporation and formation of a solid film at the resonator surface.

## 1. Introduction

The quartz crystal microbalance has in the recent past seen a tremendous spread in use and, also, a rather impressive increase in the diversity of applications [1]. In part, this development was stimulated by the second-generation QCM’s (sometimes also termed “QCM-*D*” for QCM with Dissipation monitoring [2]). These instruments supply information beyond gravimetry [3]. They do so by reporting the resonance bandwidth in addition to the resonance frequency and, also, by determining frequency and bandwidth on a number of different overtones. When applied to the study of thin films, this information can be exploited to make a statement about the sample’s softness [4]. In this regard, the QCM is superior to the optical techniques of label-free sensing, most notably surface plasmon resonance (SPR) spectroscopy [5]. SPR spectroscopy otherwise (at least today) has a superior limit of detection (LOD) and less baseline drift.

A certain problem with the second-generation QCMs is time resolution. It is difficult to determine the resonance frequency in a time of less than 100 milliseconds. Common is a data acquisition rate of 1 s^−1^. There are numerous fast processes at interfaces (such as the impact and the detachment of particles, the rupture of membranes, or double-layer charging in dynamic electrochemistry [6]), which are not easily studied with the QCM because of speed limitations.

In a previous publication, we have elaborated on the limits of a QCM’s speed and reported on an instrument, which systematically carries the QCM to these limits [7]. The instrument achieving this improved time resolution is the “multi-frequency lockin amplifier”, MLA. The MLA was developed with the aim to quickly monitor the resonances of AFM cantilevers. The inventors emphasize nonlinear behavior, which in this context implies that the resonance frequency and the resonance bandwidth depend on amplitude [8]. While such effects certainly exist for quartz resonators, they are negligible at the driving amplitudes employed here and are outside the scope. This work simply exploits parallel detection on many channels. The MLA applies up to 32 sine waves to the device under test, in parallel. There is a corresponding set of detection channels. The MLA determines the Fourier components of the input to the detector at up to 32 frequencies, which are configured (here) to coincide with the excitation frequencies. We term this mode of data acquisition a “comb measurement”. The resonator is wired such that the detector essentially determines the current through the device at the respective frequencies. Dividing by the voltage of excitation, one obtains a set of 32 complex electrical admittances *Y*(*f*_i_) with *i* labeling the different frequencies (see Equation (3)). Resonance frequencies and resonance bandwidths are obtained by fitting resonance curves to these data sets. Basically, the algorithm is equivalent to impedance analysis, the only difference being that combs are applied rather than frequency sweeps. More details on the MLA and its relation to conventional impedance analysis [9] and ring-down [2] are provided in Reference [7].

In the time domain, the combs constitute sequences of electrical pulses, spaced in time by an interval of Δ*t*_comb_ = 1/δ*f*_comb_ where δ*f*_comb_ is the frequency spacing between the members of the comb. For that reason, the time resolution of the comb measurement is 1/δ*f*_comb_. In order to catch the resonance, the frequencies must be spaced from each other by less than the bandwidth of the resonance. The resonators employed in this work had a resonance bandwidth (half-width at half-height) of 200–500 Hz, depending on overtone order. The frequency spacing of the combs was chosen as 100 Hz, which puts the time resolution to 10 milliseconds.

**Figure 1:**
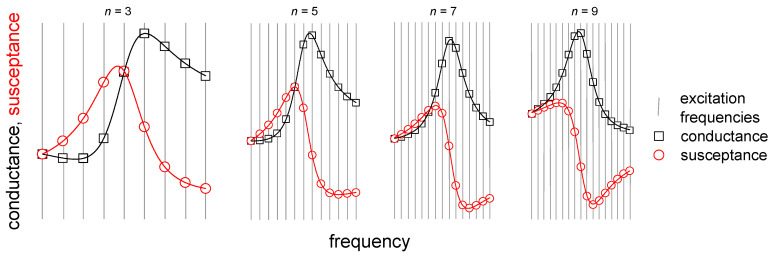
The principle of measurement. The electrical admittance is interrogated on 32 channels at the same time, which are distributed over 4 overtones (at 15, 25, 35, and 45 MHz). *n* is the overtone order.

The existing second-generation QCMs access the different overtones sequentially. That is a problem when the softness of the sample is to be inferred from the comparison between overtones. One then assumes Δ*f*(*n*) and ΔΓ(*n*) to be functions of *n*, only. If the different overtones are accessed one after the other and if, further, the properties of the sample drift in time, an apparent overtone-dependence of Δ*f* and ΔΓ may, in reality, be caused by drift. A key novelty of this work is that the 32 frequencies of interrogation have been distributed over four overtones (at 15, 25, 35, and 45 MHz, see Figure 1). An “overtone” here denotes an acoustic eigenmode. Overtones are labeled by the number of nodal planes parallel to the surface of the disk, *n* (*n* = 3, 5, 7, and 9 here). The fact that the parallel interrogation of four overtones succeeds is far from trivial. There might be cross-talk. Such cross-talk has been seen in other cases. Coupling between modes gives rise to the “activity dips”, much feared in time-and-frequency control [10]. Mode coupling was not observed here. Mode coupling is absent if the resonances are spaced widely from each other in frequency, if the mode shapes are sufficiently different, and if the nonlinearities are sufficiently small.

Parallel interrogation of modes is common practice in time-and-frequency control [11]. The “temperature-compensated crystal oscillators” (TCXOs) oscillate at both the fundamental and the 3rd overtone. TCXOs exploit the dependence of temperature-frequency-coupling on overtone order. The driving electronics infers the resonator’s temperature from the difference in the behavior of overtones 1 and 3 and uses this information to correct the clock-frequency for temperature-effects. As the name says, the TCXOs contain oscillator circuits. The problems with those are well known (capacitance compensation, the influence of damping on the oscillation frequency, and others) [12]. Interestingly, Ferrari and Ferrari have applied such a device to droplet impact (as we did, see below) [13]. The droplets contained sugar, which remained on the substrate after drying. Ferrari and Ferrari report on a transient response, but the time resolution was 2 s, while it was 10 milliseconds here.

The performance of the multi-overtone QCM was demonstrated with a study of inkjet-printed droplets. The experiment as such is simple; droplets deposited onto a QCM just about always shift the resonance frequency and the bandwidth. Early work along these lines was published by the Ward group [14]. Reference [14] interprets QCM data in terms of wetting kinetics. Droplet spreading on structured surfaces (on the macroscale) was also studied in References [15,16]. QCM-based studies of droplet drying on the macroscale (volumes > 1 µL) have been reported in References [17,18,19,20].

**Figure 2:**
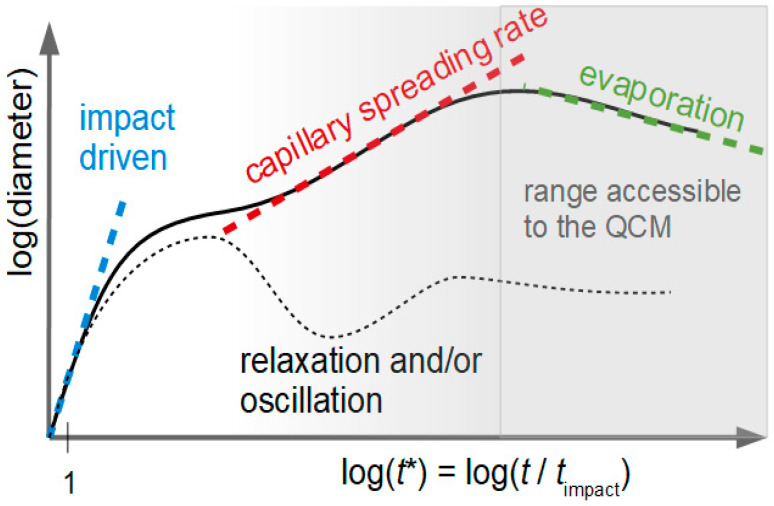
Different phases of the droplet deposition process. The immediate impact happens on the time-scale of a few microseconds. Inertial forces dominate, but viscosity and surface energy synergistically prevent splashing. After the impact, the drop may or may not undergo oscillations in shape. There usually is some further spreading, meaning that the contact line moves outwards. If the contact line in the final state is pinned, the droplet radius eventually stays constant. Otherwise, the droplet will shrink in diameter. Droplet drying may occur within less than a second. This was adapted from Reference [26] and slightly modified to include drying and the time range accessible to the QCM.

Droplet dispensing is key to a range of technologies, including 3D-printing [21], bioprinting [22], and microfluidics [23]. Even vesicles [24] and bacteria spores [25] have been printed. Because the printing device employed was of the drop-on-demand type, we limit the discussion to this technique. While inkjet printing is a flexible technique in many ways, there are certain constraints. For instance, the droplet volume usually is in the picoliter range, dictated by the application. The droplet velocity is around 10 m/s, dictated by the need to separate the droplet from the nozzle. A large-enough velocity ensures a large-enough momentum of the drop to overcome surface tension at the nozzle. These two parameters (diameter: some tens of micrometers, velocity: ~ 10 m/s) limit the duration of the impact to a few microseconds. It is convenient to define a normalized time t* as *t*/*t*_impact_ (Figure 2).

As discussed in Reference [26], the need to avoid splashing and satellite droplets imposes a constraint on what is called the Ohnesorge number *Oh*. *Oh* is defined as *Oh* = η/(γρ*a*)^1/2^ with η the viscosity, γ the surface energy, ρ the density, and *a* the droplet’s characteristic length. *Oh* should be larger than 1 but not much larger than 1. Some viscous dissipation is needed to avoid splashing but the viscosity should be as small as possible, otherwise. The second constraint concerns the Weber number *We* = ρ*av*^2^/γ with *v* the velocity of the droplet. A suitable choice is *We*~100. Inertial forces must be strong enough to deform the drop from a sphere to a hemisphere on impact.

The immediate impact is followed by droplet spreading, driven by capillary forces. During this time, the droplet shape may or may not oscillate in shape. (In the experiments reported below, the QCM-data give no hint of such oscillations.) With regard to spreading, analytical theory (sometimes attributed to Tanner [27]) predicts the droplet radius to scale as *t*^1/10^ under certain conditions. In Reference [26], the droplet radius eventually reaches a constant value (Figure 2). Motivated by the experiments reported below, we have depicted this line with a negative slope because evaporation does have an influence at these times.

Interestingly, the rate of evaporation is not discussed much in Reference [26]. The rate of evaporation (often “*E*”) is a critical parameter in the theory of film formation from latex dispersions, which has numerous analogies to droplet drying [28]. That the rate of evaporation is of some importance to inkjet printing, and can be inferred from the use of “humectants” in ink formulations [29]. Humectants (such as ethylene glycol and 2-pyrrolidone, see below) slow down evaporation. The evaporation rate is of concern if the liquid needs to infuse a porous substrate (such as paper, see Reference [30] and many others). Evaporation also is of prime importance in those cases, where the liquid is loaded with solid particles to some appreciable extent. The solids content typically is moderate because the viscosity would otherwise exceed the limits imposed by the droplet formation process. Still, when solids are contained in the ink, the drying process includes the many facets of “film formation” [28,31]. In this context, the drying rate affects skin formation [28], the coffee-stain effect [32], Marangoni convection [31], and particle deformation. Drying is, for instance, addressed in the review by Derby. In the main text, we only discuss dyes which are molecularly dissolved in the liquid. In the supporting information, we show data taken on droplets loaded with gold nanoparticles. Clearly, these behave in a more complicated way. There are discontinuities, which may be related to crack formation. These data are meant to emphasize the potential usefulness of the QCM. No attempt is made to interpret these data traces in detail.

A few side remarks concern inkjet printing in analytical chemistry. Picoliter-dosing is common in analytical chemistry because it saves resources and increases throughput. The sample volume is particularly critical for bio-molecules. Inkjet printing can be helpful in elemental analysis [33,34,35,36]. Thermal inkjet printing has been used in X-ray fluorescence analysis (XRF). Picoliter droplets have been explored for calibration of microscopic sample deposits in the analysis of atmospheric aerosols [37] as well as in semiconductor analysis [38,39]. The micro deposits out of standard solutions all have a similar shape [40]. For that reason, they can be used to investigate the physical interactions of X-rays with the residues (which includes absorption effects in total reflection X-ray fluorescence analysis (TXRF) [41]) and the performance of novel optics [42]. With known concentration ratios of elements, matrix-free relative sensitivities of a TXRF device were determined in Reference [43]. The micro deposits can also be used for calibration of TXRF devices with laser ablation combined with inductively coupled plasma mass spectrometry (ICP-MS) [33,44]. Cartridges and prototypes generating picoliter droplets are quite useful as aerosol generators in ICP-MS. Standard solutions can be sprayed with this aerosol generator directly into the ICP-MS, resulting in better sensitivities and lower detection limits [34,45].

A measurement protocol exploiting small droplets has also been proposed for the QCM in Reference [46]. If the analyte to be studied with the QCM is contained in a droplet (rather than in a bulk liquid, filling the entire cell), the sample volume can be small and the precision of the frequency readings improves because of the reduced damping.

The paper is structured as follows. In Section 2 we elaborate on technical issues linked to studies of drops and droplets with the QCM. There are a few subtleties, which—if ignored—can lead to erroneous conclusions. Section 3 provides details on the printing process and on data analysis. Section 4 shows experimental results, where a choice has been made to only show data, which can be compared to each other and which can be understood in a moderately simple frame. Further results, which are thought-provoking but are not easily interpreted in detail, have been deferred to the supporting information. Section 5 elaborates on experimental options for more detailed studies. This concerns improved time resolution, smaller droplets, colloid-loaded droplets, and textured surfaces.

## 2. General Remarks on the Response of the QCM to Loading with Droplets

Interpretation of QCM data obtained in experiments with droplets poses some challenges. The issues are:

- droplet weighing
- problems in the derivation of viscoelastic parameters caused by energy trapping
- the effects of capillarity
- compressional waves
- environmental effects

### 2.1. Droplet Weighing

At first glance, the QCM might appear to be well-suited to droplet weighing, given its exceptional precision. One might study the uniformity of droplet generation by weighing the droplets one-by-one. However, converting from frequency shift to mass by means of the Sauerbrey equation [47] requires rigid samples. The liquid would have to be incorporated into some kind of scaffold. Porous structures on quartz surfaces have been produced [48], but it cannot be taken for granted that imbibition proceeds fast enough. Part of the liquid might evaporate while the liquid fills the pores. An alternative is polymeric gels [49]. These swell on the time scale of milliseconds (with some slow tails in the kinetics). Their softness may be accounted for by suitable steps in the analysis. Of course, one can always let ink-loaded droplets dry out and weigh the pigments [13]. In this case, uncertainty remains with regard to the extent to which the ink has indeed dried out.

Weighing of individual droplets has also been achieved with conventional balances. These experiments drive the balances to their technical limits, but they can be done [50].

### 2.2. Problems in the Derivation of Viscoelastic Parameters Caused by Variable Energy Trapping

Rather than running the QCM in a gravimetric mode (meaning: weighing the sample), one may infer the sample’s viscosity from Δ*f* and ΔΓ. For semi-infinite Newtonian liquids, the increase in half-bandwidth, ΔΓ, equals the negative frequency shift, −Δ*f*. Both scale as (ρη)^1/2^ with ρ the density and η the viscosity. If the liquid covers only part of the resonator, one may modify the Kanazawa-Gordon relation [51,52] as

(1) $$\frac{\Delta f\;+\;i\Delta \Gamma }{f_{0}}\;=\;\frac{i}{{\pi Z}_{q}}\frac{A_{\mathrm{drop}}}{A_{\mathrm{eff},\mathrm{tot}\;}}Z_{\mathrm{liq}}\;=\;\frac{i}{{\pi Z}_{q}}\frac{A_{\mathrm{drop}}}{A_{\mathrm{eff},\mathrm{tot}\;}}\sqrt{i\omega \rho \left(\eta^{\prime }-i\eta^{\prime \prime }\right)}$$

*f*_0_ is the frequency of the fundamental, *Z*_q_ is the shear-wave impedance of AT-cut quartz, and *Z*_liq_ = (iωρη)^1/2^ is the liquid’s shear wave impedance. η = η′ − iη″ is viewed as a complex viscosity. (If η″ is nonzero, ΔΓ is larger than −Δ*f*). The term *A*_drop_/*A*_eff,tot_ accounts for the finite droplet area. *A*_eff,tot_ is the acoustically active area. It is calculated from the amplitude distribution of the transverse wave. For details, see Reference [53]. The acoustically relevant droplet area, *A*_drop_, may be unequal to the geometric area because of the nontrivial amplitude distribution.

Equation (1) looks attractive but there are some difficulties in the details. Quantitative data analysis based on Equation (1) can, in principle, be aided by an optical determination of the droplet area *A*_drop_. With known droplet size, one may invert Equation (1) for viscosity [54]. One may also, in principle, treat the term *A*_drop_/*A*_eff,tot_ as an unknown prefactor and make a statement on η′/η″ (the “loss factor” or “loss tangent”), based on the ratio ΔΓ/(−Δ*f*). However, the quantitative interpretation of the ratio ΔΓ/(−Δ*f*) and, also, of the overtone dependence of Δ*f* and ΔΓ with Equation (1), is problematic. The reasons are discussed in Reference [55]. Complications arise because a sample, which touches the resonator in the center only, increases the efficiency of energy trapping [56]. The amplitude distribution changes in response to the load and the change in energy trapping affects −Δ*f* and ΔΓ.

### 2.3. Effects of Capillarity

The question of whether the QCM is sensitive to the surface tension of a liquid-air interface has been addressed in 1994 already [14]. Basically, the answer is no. The influence of surface energy is small, as can be inferred from the capillary number *Ca* = σ_vis_/σ_cap_ = ωη/(γ/*r*). ω is the angular frequency, η is the viscosity, γ is the surface energy, and *r* is the radius of curvature. The capillary number compares viscous stress, σ_vis_, to capillary stress, σ_cap_. Unless the radius is in the nanometer range, the viscous stress is much larger than the capillary stress. Capillary stress does exert a small transverse force onto the resonator because the motion of the resonator distorts the droplet. However, these stresses are negligible for droplet sizes larger than about 1 μm. The matter is also discussed in Reference [57] (which is concerned with nanobubbles).

### 2.4. Compressional Waves

Reference [58] elaborates in some length on a problem in droplet characterization with a QCM, which occurs with droplets larger than about a millimeter. The QCM is not a pure thickness-shear resonator; there are flexural admixtures to the modes of vibration, which launch longitudinal waves into the medium under study. These propagate (shear waves do not), are reflected somewhere, and return to the resonator [59]. However, this problem is of minor influence as long as the drop height is much less than the wavelength of compressional waves. This is the case here. Compressional-wave effects are small here, as evidenced by the absence of coupled resonances, which would be associated with them [58].

### 2.5. Environmental Effects

Given that the effects amount to a few Hz only, there is a worry about environmental effects caused by variable temperature and stress. The temperature of the droplet slightly differs from the temperature of the resonator. However, the liquid volume is small here, which implies that the heat transferred across the resonator surface is correspondingly small. Heat transfer occurs on a time scale of milliseconds. This estimate is derived from τ_HT_ ≈ L^2^/D_th_ with τ_HT_ the characteristic time for heat transfer, L the characteristic length, and D_th_ the thermal diffusivity. With L ≈ 100 µm and D_th_ ≈ 10^−2^ cm^2^/s, a time of about 1 ms is obtained. The effects of stress should be small, likewise, given the droplet’s small mass.

## 3. Materials and Methods

### 3.1. Inkjet Printing

The core of the printer consists of a Q2299A mount for the cartridge (HP, Palo Alto, CA, USA). The cartridges were of the type C6602A from HP [60]. With a conventional weighing experiment using many droplets, the droplet volume was determined as 165 ± 1 pL [43,61], consistent with the specifications given by the manufacturer [60]. The mount was attached to a Newport 430 linear stage (Newport, Irvine, CA, USA) with two home-built parts (3D-printed from acrylonitrile butadiene styrene, ABS) [42]. The cartridge was controlled from an Arduino Uno Rev3 (Arduino, New York, NY, USA) and an InkShield board, supplied by N.C. Lewis (Nerd Creation Lab, Everett, WA, USA) [62].

In order to adapt the cartridge for use with the QCM, the ink and the sponge were removed, followed by cleaning with ultrapure water. The cartridges were then closed with a new lid, which had been 3D-printed. For cleaning, 30,000 droplets (~4.8 µL) were printed three times from each nozzle. This procedure was repeated twice before the cartridges were allowed to dry in air at room temperature. For every printing experiment, 2 mL of the printing fluid was filled into the chamber. The lid was sealed with tape and the entire assembly was positioned 15 mm above the QCM. The software allows us to print single droplets on demand. The rest time between two droplet ejections was twenty seconds. Every droplet was ejected from a new nozzle of the printer head. Each droplet behaves like the “first drop” (meaning that the nozzle is dry at the time of printing).

Two base fluids were used, namely ultrapure water and a mixture of 2-pyrrolidone (purum, ≥99%, Fluka, Merck, Darmstadt, Germany), ethylene glycol (p.a., 99.5%, Riedel de Haën, Honeywell, Morristown, New Jersey), and ultrapure water (resistivity ρ > 18.2 MΩ cm, Purelab Flex 4, ELGA Veolia, Paris, France). The most commonly used composition was 30:30:40 vol% 2-pyrrolidone:ethylene glycol:water (P:E:W). We term this fluid “30:30:40”. 2-Pyrrolidone and ethylene glycol have viscosities of 13.3 and 16.1 mPa s, respectively (water: 0.89 mPa s) [63,64,65]. Experiments with varied composition (P:E:W = 30:30:40, 40:30:40, 30:40:30 and 40:40:20) did not show significant differences.

Two different dyes were studied, which were fluorescein (p.a., Riedel de Haën, Honeywell, Morristown, NJ, USA) and Food Black 2/black 7894 (5 weight%, contained in the original HP ink). Dye concentration was varied, but the dependence on dye concentration was weak (see the supporting information). Stock solutions were prepared by dissolving the dye in the base fluid at a mass concentration of 5% or 10%. Ammonia was added at a concentration of 2.5 vol% to fluids containing fluorescein to stabilize fluorescein against precipitation. Also, a slightly alkaline pH prevents corrosion at the nozzle. Stock solutions were diluted to the desired concentrations before measurement.

### 3.2. Quartz Crystall Microbalance (QCM)

Gold-coated resonator crystals with a fundamental frequency of 5 MHz and a diameter of 14 mm were supplied by Quartz Pro (www.quartzpro.com). The holder was built in-house. The temperature was 22 ± 1 °C. The acoustic resonances were probed using the multi-frequency lockin amplifier (MLA) supplied by Intermodulation Products AB (Stockholm, Sweden). (Similar functionality is implemented in the unit HH2LI from ZH-instruments, Zurich, Switzerland). The time resolution was 10 milliseconds for the comb measurements. Δ*f*(*t*) and ΔΓ(*t*) were determined on four overtones at 15, 25, 35, and 45 MHz.

### 3.3. Comb. Measurements

As sketched in Figure 1, the multi-frequency lockin-amplifier (MLA) applies a set of 32 sine waves to the device. From the currents, one infers the complex electrical admittance *Y*(*f*_i_). Ideally, the electrical admittance of a resonator at the frequency *f*_i_ would be given as

(2) $${Y(f}_{i})\;=\;\frac{{i\Gamma G}_{\max}}{f_{\mathrm{res}}{\;-\;f}_{i}\;+\;i\;\Gamma }$$

*f*_res_ and Γ are the resonance frequency and the half-bandwidth, respectively. *G*_max_ is an amplitude. We are only interested in *f*_res_ and Γ (more precisely, in shifts thereof, Δ*f* and ΔΓ). Equation (2) does not cover the parallel capacitance, *C*_0_, and it does not catch imperfect calibration. The “phase-shifted Lorentzian” provides for an additional set of three fit parameters (a phase, φ, and a complex offset of the admittance, *G*_off_ *+* i*B*_off_), which account for *C*_0_ and imperfect calibration:

(3) $${Y(f}_{i})\;=\exp(i\varphi )\frac{{i\Gamma G}_{\max}}{f_{\mathrm{res}}{\;-\;f}_{i}\;+i\;\Gamma }+G_{\mathrm{off}}+{iB}_{\mathrm{off}}$$

All fit parameters (6 in total) are real numbers.

The spacing between the frequencies of the comb was chosen as 100 Hz. With 8 frequencies per overtone, the total width of each of the four combs is 700 Hz. The resonant half-bandwidths, Γ, were between 200 and 500 Hz. Further increasing the time resolution would have made the combs wider than the resonances, which would have compromised precision.

### 3.4. Data Processing and Interpretation

**Figure 3:**
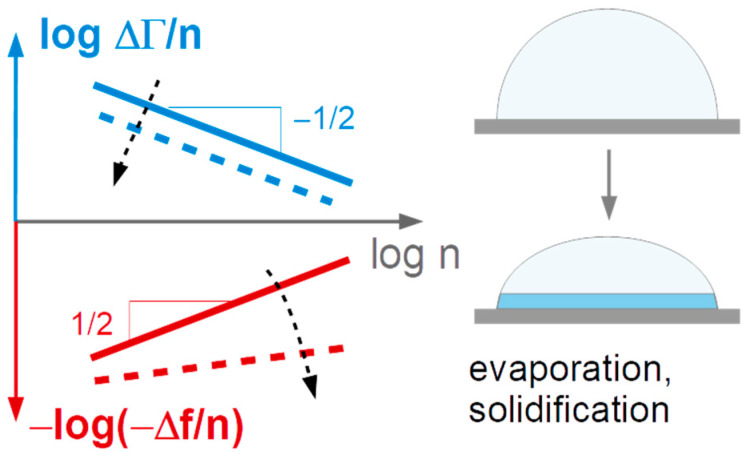
As drying proceeds, the ratio ΔΓ/(−Δ*f*) decreases, while the power law exponent of −Δ*f*(*n*) increases. This can be explained with the formation of a solid film at the substrate surface. In the limit of the dry film, the ratio ΔΓ/(−Δ*f*) goes to zero and −Δ*f* scales as *n* (not as *n*^1/2^, as in the case of the Newtonian liquid.) The deposition of solid matter at the substrate surface drives the system in this direction.

**Figure 4:**
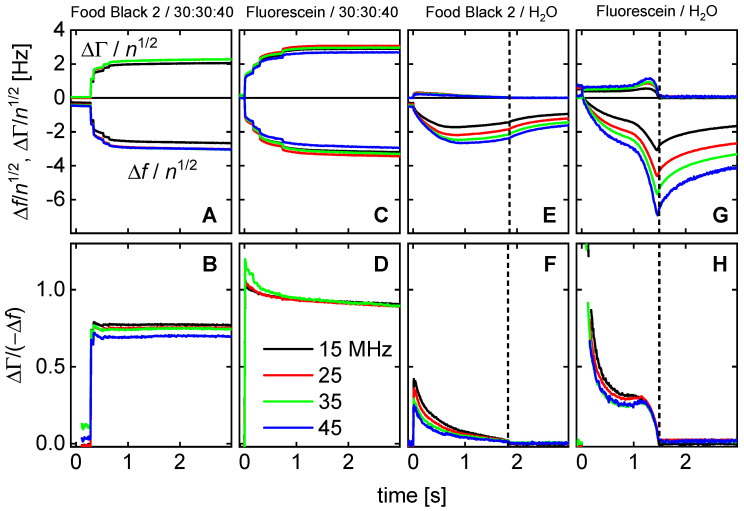
(**Top**) Shifts of frequency and bandwidth for the impact of an individual droplet from the four types of solutions indicated in the titles. Δ*f* and ΔΓ have been normalized to *n*^1/2^ because this scaling makes the data from different overtones overlap. The dye concentrations were 0.5 wt% (Food Black 2) and 1.5 wt% (fluorescein) in water and 30:30:40. For the 30:30:40-fluid, drying is slow (>20 s). Droplets based on water dry out in less than 2 s. (**Bottom**) The ratio ΔΓ/(−Δ*f*) always decreases with time.

**Figure 5:**
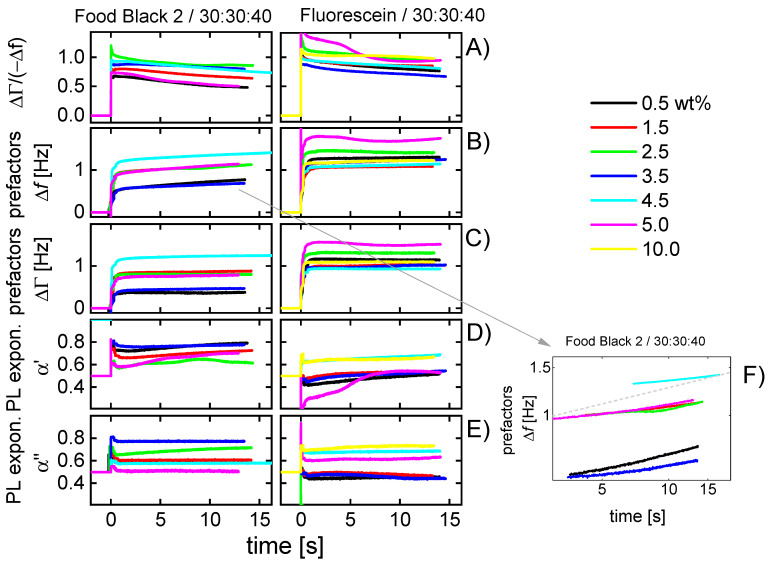
Data extracted from Δ*f*(*n*) and ΔΓ(*n*) as described in Section 3.3. The dye concentrations were between 0.5 and 10 wt%. The third droplet impact was chosen for display. (**A**) The ratio ΔΓ/(−Δ*f*) averaged over the 4 overtones. (**B**,**C**) Prefactors *A*_Δ*f*_ and *A*_ΔΓ_ obtained by fitting power laws to Δ*f*(*n*) and ΔΓ(*n*). The prefactors are related to the wetted area. (**D**,**E**) Power-law exponents α′ and α″ (see Equation (4)). The power-law exponents would be 1/2 for the semi-infinite Newtonian liquid. (**F**) The log-log plot of *A*_Δ*f*_. The dashed line indicates the prediction following Tanner’s law (droplet radius scales as *t*^1/10^, droplet area scales as *t*^1/5^).

Given the difficulties discussed in Section 2.2, we do not attempt to quantitatively derive materials parameters from Δ*f*(*t*) and ΔΓ(*t*), but rather interpret the time evolution of Δ*f* and ΔΓ on a heuristic basis. We aggregate the data as follows:

- Power laws are fitted to the functions −Δ*f* and ΔΓ versus *n*:
  (4) $${-\Delta f\;=\;A}_{\Delta f}\;n^{\alpha^{\prime }}\;{\Delta \Gamma \;=\;A}_{\Delta \Gamma }n^{\alpha^{\prime \prime }}$$
  The prefactors *A*_Δ*f*_ and *A*_ΔΓ_ are related to the droplet area. The power-law exponents report intrinsic properties, independent of droplet size. There are two limiting cases. For the semi-infinite Newtonian liquid, one expects α′ = α″ = 1/2 (see Equation (1)). For solid films, one expects α′ = 1 and α″ = 0. Immediately after impact, we find α′ ≈ α″ ≈ 1/2. Deviations (which are present) may come about by energy trapping, by compressional waves, by capillarity, by relaxation processes with rates comparable to the frequency of vibration, and by the formation of a solid film at the substrate surface (see Figure 3).
- We plot the ratio ΔΓ/(−Δ*f*) versus time (Figure 4 and Figure 5). ΔΓ/(−Δ*f*) is an indicator of energy dissipated per unit area and unit time.

## 4. Results and Discussions

**Figure 6:**
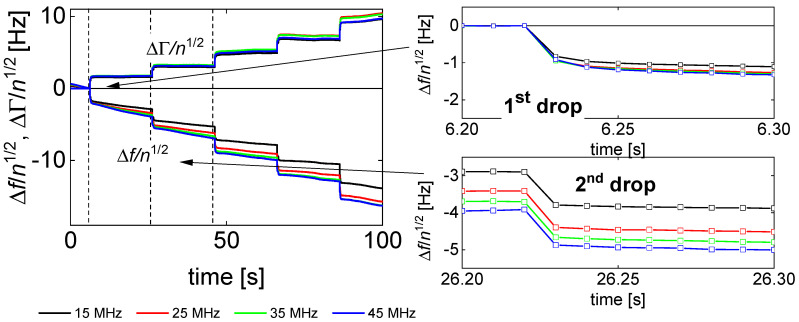
(**Left**) Raw data from an experiment with Food Black 2 in the liquid 30:30:40 (concentration: 10 wt%), where 10 droplets were deposited at separate spots. Only 5 events are shown for clarity. Data have been normalized to *n*^1/2^ because this lets the data from the different overtones overlap. (**Right**) Impact no 2 shifts Δ*f* slightly less than the previous impact no 1.

In the first step, 12 droplets were sequentially deposited onto different spots on the resonator surface. Figure 6 shows an example. (It shows 5 depositions only for the sake of clarity.) Clearly, there is some variability between the different droplets. Potential reasons are:

- Variable conditions of droplet formation at the nozzle. The print head has 12 nozzles, which were actuated one after the other. Every droplet was a “first droplet” in the sense that the nozzle was dry. First droplets sometimes behave less reproducibly than droplets ejected after some running-in. The distance between two nozzles at the printer head was 250 µm. Due to evaporation inside the cartridge, the droplets may increase slightly in droplet mass (following slight increases in dye concentration).
- There may be differences in substrate microstructure and substrate wettability on the micro-scale. No attempts were made to avoid these.
- The amplitude distribution of the QCM often displays some irregular variability over the area of the plate. It goes back to defects in the crystal, which are not visible to the eye. An image is shown in Reference [66]. The local amplitude affects the magnitude of −Δ*f* and ΔΓ.
- Apart from the small-scale variability in amplitude, there is a wafer-scale variability, often modeled as a Gaussian [67]. While the Gaussian is of influence, in principle, the total spread of droplet positions is 2.25 mm (corresponding to a line of 10 droplets, spaced by 250 µm). The length of this line is to be compared to the electrode radius of 3.5 mm. Presumably, the small-scale variability in amplitude (often disregarded) is more important than the Gaussian.

Figure 4 shows shifts of frequency and bandwidth (top) and the ratios ΔΓ/(−Δ*f*) (bottom) for the four experiments chosen to be discussed in more depth. These data pertain to the third droplet. Figure 4 only shows data from the first three seconds after the impact of an individual droplet. (Figure 5 extends the time range to 15 s).

Dashed vertical lines denote the time when the drop has converted from a liquid state to a solid state.

**Figure 7:**
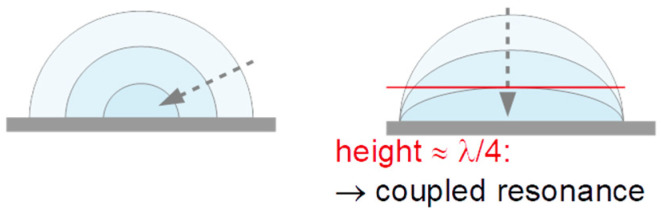
Two modes of droplet drying. On the left-hand side, the three-phase line is free to move, keeping the contact angle constant. On the right-hand side, the three-phase line is pinned. This latter scenario leads to maxima in −Δ*f* and ΔΓ, when the height of the drop is about a quarter of the wavelength of the shear sound. The wavelength of the shear sound is close to the penetration depth of the shear wave, which is around 100 nm, depending on the viscosity and overtone order. Maxima in −Δ*f* and ΔΓ are observed in the kinetics of drying of fluorescein in water (Figure 4G).

The results displayed in Figure 4A–D can be interpreted as follows:

−Δ*f* and ΔΓ are about equal in magnitude. Equal magnitudes are expected for semi-infinite Newtonian liquids. These samples do not strongly deviate from this expectation.
- Data from different overtones overlap if they are divided by *n*^1/2^. Again, square-root-*n*-scaling is expected for the Newtonian liquid.
- Spreading proceeds further after the impact. This is shown by the jumps at *t* < 1 s after impact in Figure 4A,C. There is a further gradual increase in longer time scales (also see Figure 5B,C).
- The ratio ΔΓ/(−Δ*f*) decreases, where the decrease is slightly stronger than average in the first few fractions of a second. Presumably, this initial decrease is related to microscale wetting.
- The results displayed in Figure 4E–H can be explained as follows:
- There are rather strong differences in the behavior of droplets printed from the liquid 30:30:40 (2-pyrrollidone: ethylene glycol: water) and droplets printed from aqueous solutions. These liquids mostly differ in the evaporation rate. A second difference is the viscosity, which is about a factor of 10 higher for the 30:30:40-liquid than for water.
- The droplets printed from aqueous solutions dry in less than 2 s. After drying, the bandwidth reverts to its original value. The frequency shift does not, because the dye remains on the substrate and acts as a Sauerbrey-type sample. The dye is moist, initially, and loses humidity later, as evidenced by a gradual increase in Δ*f*.
- There is an interesting peculiarity for fluorescein in water (Figure 4G,H). For this sample, the bandwidth goes through a maximum shortly before returning to its base value before impact. This behavior would typically be associated with a film resonance, as sketched in Figure 7. If the three-phase line is pinned, the droplet will turn into a “pancake” while drying. When the height equals a quarter of the wavelength sound (about equal to the penetration depth of the shear wave, ~100 nm), the film itself is a resonator with a frequency equal to the frequency of the quartz crystal. At this point, the bandwidth goes through a sharp maximum and the frequency also shows a characteristic pattern. The drying kinetics shown Figure 4E,G suggest that the contact line is pinned for fluorescein, while it is not pinned for Food Black 2. This difference in phenomenology is seen for all 10 droplets, not just for the third droplet (the drying kinetics of which is displayed here.)

Figure 5 shows parameters derived from −Δ*f*(*n*) and ΔΓ(*n*) as described in Section 3.3, namely *A*_Δ*f*_, *A*_ΔΓ_ as well as α′ and α″. The time axis has been extended to 15 s. The graph now includes a significant portion of the drying process. Only data from droplets made from the fluid 30:30:40 are shown because these are more typical for the technical process than the aqueous solutions. Different concentrations of dye in the range of 0.5–10 wt% were printed. Again, the data of the third droplet was used to display the results.

The results can be summarized as follows:

- ΔΓ/(−Δ*f*) decreases not only in the first few 100 milliseconds (Figure 4B,D,F,H), the decrease continues slowly over the entire drying time.
- The power-law exponents of −Δ*f*(*n*) (see Equation (4)) increase with time. Both the decrease in ΔΓ/(−Δ*f*) (Figure 6A) and the increase in α′ (Figure 5D) can be explained by the formation of a solid film at the resonator surface as sketched in Figure 3. When a film forms at the substrate surface, this film adds a Sauerbrey-type contribution to the overall shifts of frequency and bandwidth. Accordingly, the bandwidth shifts become less pronounced compared to the frequency shift. The power-law exponent in fits of power laws to −Δ*f*(*n*) shifts towards 1 (which is the exponent expected for dry films).
- The droplet area slowly increases over the entire drying time (Figure 5B,C). Figure 5F shows a log-log plot of *A*_Δ*f*_ versus time. The dashed line indicates the slope, which would be expected from Tanner’s law [26]. Tanner’s law states that the droplet radius should scale as *t*^1/10^, which implies that the droplet area scales as *t*^1/5^.
- The dependence of the drying kinetics on dye concentration is comparable in magnitude to the drop-to-drop variability. The respective graph has been moved to the supporting information (Figure S1). This is not meant to say that the observed variability (which includes the peculiar behavior of the 5%-sample) was not systematic. It might be systematic, but interpreting the limited amount of data shown in Figure 5 would amount to speculation.

Again, this discussion was limited to examples that can be understood easily. The supporting information shows the data from two more experiments, the results of which one gets not easily interpreted.

## 5. Future Directions

In order to advance studies of droplet-drying with a QCM, the following experimental and instrumental aspects may be considered in the future:

- The droplet volume (160 pL) was on the large side as far as common technical procedures are concerned. The sensitivity would easily suffice to also study droplets with volumes of a few tens of pL. If sensitivity is a problem and if the droplet volumes should go down to a few pL, one may consider the use of HFF-resonators (high-frequency-fundamental-resonators) [7,68]. These consist of a thin quartz membrane in a thicker frame. The recess is created with reactive-ion etching. These resonators are exceptionally sensitive and they have a small active area. Overtones are not easily accessed, though. (Arcamone et al. have studied femtoliter droplets with a nanomechanical resonator [69], but such studies would not have been possible with a conventional QCM.)
- Smaller droplets would dry faster, and the benefits of the QCM-based measurement would play out stronger.
- Time resolution can be improved to about 100 µs with single-frequency measurements, as discussed in Reference [70]. Presumably, this would allow access to the time scale, where droplets oscillate in shape.
- Combination with a high-speed camera is worth consideration to study droplet impact and evolution on the surface with an independent method [71].
- It would be interesting to study textured or porous substrates. Those would have to be rigid and thinner than a few microns. Nonetheless, they can be applied to the QCM-surface as films by printing as well.
- Colloid-loaded droplets (see Figure S2 in the supporting information) will reveal details of the film formation process.

## 6. Conclusions

The performance of a fast multi-overtone QCM was demonstrated, which is well suited to study transient effects in inkjet printing. The time resolution is 10 milliseconds. The immediate impact (occurring on the time scale of a few tens of µs) is not resolved. Droplet impact is followed by:

- a fast decrease in ΔΓ/(−Δ*f*), indicative of microscale wetting, in general.
- a slow, further decrease in ΔΓ/(−Δ*f*), paralleled by an increase in the power-law exponent of –Δ*f*(*n*), which can be explained by the formation of a solid film at the resonator surface for the 30:30:40 samples.
- a slow, further spreading over a time scale of a few seconds, following Tanner’s law for the 30:30:40 samples.

The experiments are not at all demanding. They can be easily extended to other processes with characteristic times in the millisecond range.

## Supplementary Materials

The following are available online at https://www.mdpi.com/1424-8220/20/20/5915/s1, Figure S1: Derived parameters as shown in Figure 6 in the main text as a function of dye concentration, Figure S2: The impact of a droplet containing gold nanoparticles, Figure S3: Impacts of macroscopic droplets (2 µL).
