# Supplementary material for: A Quartz Crystal Microbalance, Which Tracks Four Overtones in Parallel with a Time Resolution of 10 Milliseconds: Application to Inkjet Printing
Source: Sensors (Basel). 2020 Oct 20;20(20):5915. doi: 10.3390/s20205915 (PMC7589769; doi:10.3390/s20205915)
Supplement: Supplementary file 1 [file sensors-20-05915-s001.pdf]

sensors

Supporting Information

A Quartz Crystal Microbalance, which Tracks Four Overtones in Parallel with a Time Resolution of 10 Milliseconds: Application to Inkjet Printing

Christian Leppin [1], Sven Hampel [2], Frederick Sebastian Meyer [1], Arne Langhoff [1], Ursula Elisabeth Adriane Fittschen [2], and Diethelm Johannsmann [1,*]

[1] Institute of Physical Chemistry, Clausthal University of Technology, Arnold-Sommerfeld-Str. 4, D-38678 Clausthal-Zellerfeld, Germany; christian.leppin@tu-clausthal.de (C.L.); frederick.sebastian.meyer@tu-clausthal.de (F.M.); arne.langhoff@tu-clausthal.de (A.L.).

[2] Institute of Inorganic and Analytical Chemistry, Clausthal University of Technology, Arnold-Sommerfeld-Str. 4, D-38678 Clausthal-Zellerfeld, Germany; sven.hampel@tu-clausthal.de (S.H); ursula.fittschen@tu-clausthal.de (U.F).

* Correspondence: johannsmann@pc.tu-clausthal.de

Received: date; Accepted: date; Published: date

1. Dependence of derived parameters (amplitudes, power-law exponents) on concentration

In the main text, it was stated that the dependence of drying kinetics on the dye concentration was weak. It is weak, but it is not strictly absent. Figure S 1 shows these data.

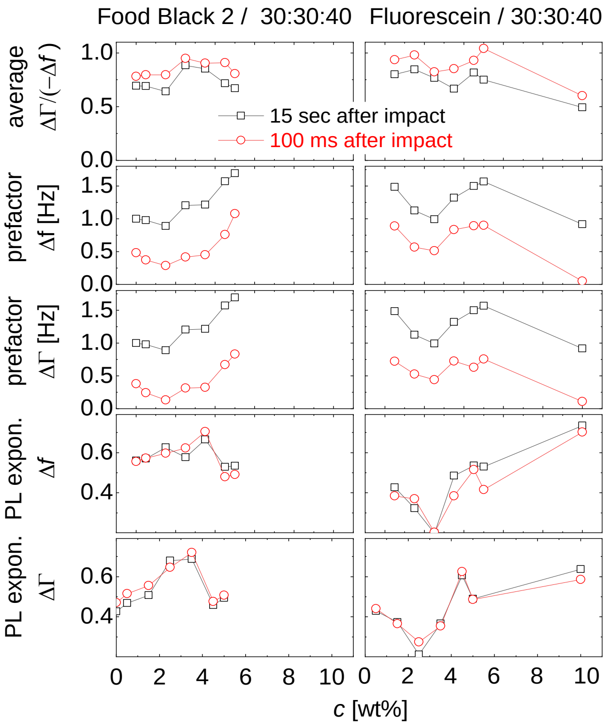

Figure S 1. Derived parameters as shown in Figure 5 in the main text as a function of dye concentration. Circles and squares correspond to times shortly after the impact and 15 seconds after impact, respectively. Data were averaged over all 10 droplets to improve statistics.

2. Experiments with gold nanoparticles

Several experiments were undertaken with more complicated samples. The results are not easily interpreted. One such experiment concerns a liquid loaded with colloids (gold nanoparticles, concentration 0.5 mM). Colloidal dispersions go through what is called the film-formation process [72], while they dry. Film formation may entail skin formation, cracking, the coffee-stain effect, Marangoni convection, and other peculiarities. The drying kinetics of drops printed from a liquid loaded with gold nanoparticles (Figure S 2) clearly reveals complications. Possibly, the discontinuities are related to cracking.

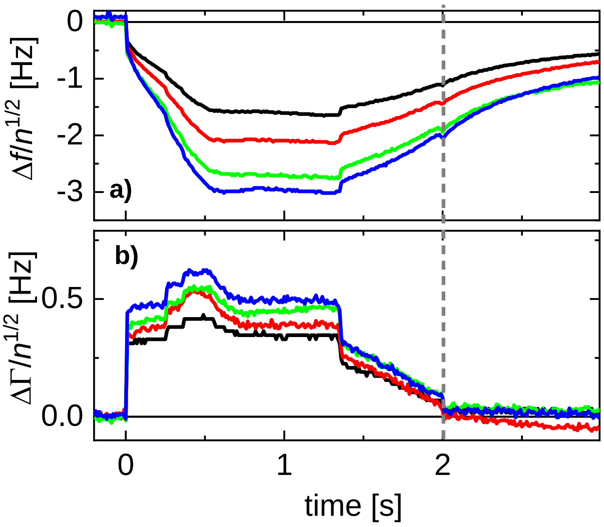

Figure S 2. The impact of a droplet containing gold nanoparticles. Clearly, the phenomenology is complicated. The discontinuities might be caused by crack formation.

Gold nanoparticles were produced by a slightly modified Turkevichs method [73]. A 5 mM chloroauric acid ($H[AuCl_4]$) solution with a volume of 250 mL was prepared by dissolving a gold foil (Au, 99.99%, Sigma-Aldrich, St. Louis, MO, USA) in aqua regia. 2.5 mL of this solution was diluted to a concentration of 0.5 mM by adding ultrapure water (resistivity ρ > 18.2 MΩ cm, Purelab Flex 4, ELGA Veolia, Paris, France). Before stirring the solution under reflux for 10 min, the pH was adjusted to 4.99 at 82.2 °C by adding 0.1 M sodium hydroxide (≥99.0%, Merck, KGaA, Darmstadt, Germany) using an automated titrator (TitroLine 7000, Xylem Analytics Germany Sales GmbH & Co. KG, Weilheim, Germany). During reflux, 250 µL of a 750 mM sodium citrate (≥99.5%, Sigma-Aldrich, St. Louis, MO, USA) and 250 µL of a 0.24 µM tannic acid solution (≥99.0%, Merck KGaA, Darmstadt, Germany) were added. Immediately after addition, the solution turns red. After keeping the solution under reflux for 5 more minutes, the solution was stored at 2 °C. The hydrodynamic diameter was determined by DLS (Zetasizer Nano ZS, Malvern Instruments Limited, Malvern, United Kingdom) as d_H = 17.3±5.9 nm.

3. Experiments with macroscopic drops

Figure S 3 shows drying experiments undertaken with macroscopic drops (volume: 2 µL) deposited onto the plate from a height of about 1 cm. The velocity at impact was about 0.2 m/s. The panels at the top, the center, and the bottom show the drying of water, of the liquid 30:30:40, and of a polymer solution, which is very viscous (a 10 000 ppm aqueous solution of sodium acrylic acid, NaPAA, $M_W \approx$ 15 000 g/mol, Sigma Aldrich, St. Louis, MO, USA). For the water and the liquid 30:30:40 the impact is still not resolved, although the droplet diameter has increased and the velocity has decreased. The impact *is* revolved for the very viscous drop. Viscosity slows the formation of the contact down to the extent that it can be followed by the QCM.

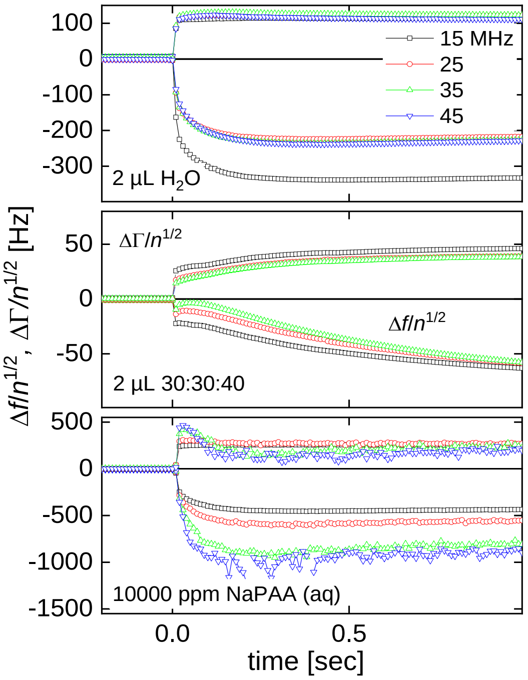

Figure S 3. Impacts of macroscopic droplets (2 µL). The liquid was dropped manually from a height of about 1 cm, leading to a velocity of ~0.2 m/s.
